# Prognostic plasma biomarkers of early complications and graft-versus-host disease in patients undergoing allogeneic hematopoietic stem cell transplantation

**DOI:** 10.1002/jha2.26

**Published:** 2020-06-17

**Authors:** Balaji Balakrishnan, Raveen Stephen Stallon Illangeswaran, Bharathi M Rajamani, Aswin Anand Pai, Infencia Xavier Raj, Daniel Zechariah Paul, Kavitha Lakshmi, Thenmozhi Mani, Ezhilpavai Mohanan, Uday Kulkarni, Anup Joseph Devasia, NA Fouzia, Anu Korula, Aby Abraham, Alok Srivastava, Vikram Mathews, Sophie Paczesny, Biju George, Poonkuzhali Balasubramanian

**Affiliations:** 1 Department of Haematology, Christian Medical College, Vellore, India; 2 Department of Biostatistics, Christian Medical College, Vellore, India; 3 Indiana University School of Medicine, Indianapolis, Indiana, USA

**Keywords:** GVHD, HSC transplantation, prognostic

## Abstract

Early complications post hematopoietic stem cell transplantation (HSCT) such as sinusoidal obstruction syndrome (SOS) and graft versus host disease (GVHD) can be life threatening. Although several biomarkers have been identified to correlate with these complications and their response to treatment, these are yet to be used in clinical practice. Here, we evaluated circulating endothelial cells (CECs) (n = 26) and plasma biomarkers (ST2, REG3*α*, VCAM1, ICAM1, TIM3) (N = 210) at early time points, to determine their association with early complications post-HSCT. Elevated CEC counts at the end of conditioning was associated with GVHD, indicating endothelial damage during HSCT. Plasma levels of REG3*α*, VCAM1, ICAM1, and TIM3 on day 14 (D14) and D14 ICAM1 and D28 ST2 were significantly higher in patients with SOS and aGVHD, respectively. Upon sub-group analysis, D28 ST2, D14/D28 REG3*α*, and D14ICAM1 levels were significantly higher in patients with gastrointestinal GVHD, while D28ST2 was higher in those with skin/liver GVHD. High ST2 levels on D28 was significantly associated with non-relapse mortality (NRM) and overall survival. Our results suggest that elevated ST2 levels on D28 could predict the likelihood of developing aGVHD and could influence NRM and OS.

## 1 Introduction

Hematopoietic stem cell transplantation (HSCT) is an established curative treatment option for both hematological malignancies and non-malignant diseases [1,2]. However, the success of HSCT is limited by several complications including regimen related-related toxicities (RRT) such as sinusoidal obstruction syndrome (SOS) [3], mucositis, and graft-versus-host disease (GVHD) [4], which could be life threatening. Minimally invasive tests such as plasma or cellular biomarkers could be useful in the diagnosis, prognosis, and response to therapy of these complications.

Biomarkers could be either: (a) diagnostic biomarker (that identifies patients at the onset of clinical disease); (b) prognostic (that identifies the likelihood of a clinical event occurrence in HCT recipients), or (c) predictive (that categorizes patients by their likelihood of response to a particular treatment when measured prior to the treatment) [2]. Since GVHD and RRTs share a common trigger, particularly damaged endothelium [5], circulating endothelial cells (CECs) have been evaluated as a diagnostic biomarker for SOS [6] and as a prognostic marker for endothelial damage [7–10], acute GVHD [7,11], and thrombotic microangiopathy (TMA) [12]. Similarly, regulatory T cells (Tregs) [13], CD146^+^ T cells [14] and invariant natural killer T cells [15] have been evaluated as diagnostic/prognostic biomarkers for acute GVHD.

Several groups have used proteomics strategy to discover candidate plasma biomarkers that were found to be associated with: (a) GVHD onset (interleukin-6 [IL6], STimulation-2 [ST2], T cell immunoglobulin mucin 3 [TIM3], Regenerating islet-derived protein 3 alpha [REG3*α*]); (b) GVHD severity (IL6, ST2, elafin, TIM3); (c) response to GVHD therapy (ST2, TIM3, REG3*α*, amphiregulin); (d) non-relapse mortality (NRM) (IL6, ST2, REG3*α*, TIM3, amphiregulin, elafin); (e) skin specific GVHD (elafin); (f) gastrointestinal GVHD (REG3*α*, TIM3), and (g) diagnosis of SOS (ST2, angiopoietin-2 [ANG2], hyaluronic acid [HA], l-ficolin, and vascular cell adhesion molecule-1 [VCAM1]) [16,2].

However, a vast majority of the studies have only reported diagnostic biomarkers for HSCT complications and not as prognostic biomarkers. Also due to the different sources of reagents/methods for measuring these biomarkers, establishing cut-off levels for clinical use need to be established in individual laboratory. Here, we evaluated both cellular and soluble plasma biomarkers from preconditioning until D28 post-HSCT to assess markers of endothelial damage and to evaluate their potential as prognostic values for GVHD and early transplant related complications.

## 2 Patients and Methods

### 2.1 Study design

Patients undergoing HSCT for various hematological disorders at the department of Haematology at the Christian Medical College in Vellore, India, were enrolled in the study after obtaining written informed consent/assent from the patients (or parents in case of children below the age of 12 years). This study protocol was approved by Institutional Review Board (IRB No: 9411 dated 29-04-2015). Between March 2016 and March 2019, blood samples were collected from patients at various time points, which included preconditioning, end of conditioning, D14, and D28 post-HSCT samples. Cellular biomarkers (CECs) were evaluated by flow cytometry and plasma were stored at −80°C for soluble biomarkers measurements by ELISA. Subsequently, samples were only stored for plasma biomarkers measurement. The frozen plasma samples once thawed were analyzed for all five analytes (ST2, REG3*α*, VCAM1, ICAM1, and TIM3) at the same time to avoid variations due to repeated freeze-thaw cycles. All assays were performed at the central laboratory of the department of Haematology, Christian Medical College, Vellore.

### 2.2 Sample preparation and processing

Nine milliliters of whole blood was obtained from patients at indicated time points in heparin containing vacutainers (Becton Dickinson, Franklin Lakes, and NJ). Plasma was separated immediately by centrifugation at 4000 rpm for 5 min at 4°C and stored at −80°C until use. The cells were used for flow cytometric evaluation of CECs.

### 2.3 Antibodies and ELISA kits

The antibodies CD31-FITC, CD45-PerCP, and CD146-PE were purchased from BioLegend (San Diego, CA, USA) and CD133-APC from eBiosciences (San Diego, CA, USA). Hoechst 33342 stain was obtained from Sigma (St. Louis, MO, USA). The ELISA kits used were ST2 (DST200; R&D Systems Quantikine®, Minneapolis, MN, USA), VCAM1, ICAM1, and TIM3 (DY809, DY720, and DY2365; R&D Systems Duosets®, Minneapolis, MN, USA) and REG3*α* (Cat. No. 5323; Ab-Match Assembly Human PAP1 [REG3*α*] kit, MBL International Corp, Japan).

### 2.4 Flow cytometric evaluation of CEC

Flow cytometric evaluation of CECs was performed as described previously. [17] Briefly, red blood cells from whole blood were lysed using ammonium chloride potassium (ACK) lysis buffer. The cells were then washed with phosphate buffered saline (PBS), incubated with conjugated antibodies. Hoechst 33342 stain was used to identify nucleated cells and to exclude platelets and microparticles. Samples were acquired on Gallios (Beckman Coulter) flow cytometer with a maximum of 1 x 10^6^ events. Data analysis was done using Kaluza software (Beckman Coulter). The definition of a CEC was Hoechst 33342^+^/CD45^−^/CD31^+^/CD146^+^/CD133^−^. CEC numbers were normalized to total white cell count as: CEC numbers = CEC% x WBCs/mL. Log_10_ transformed values were then used for analysis owing to skewed raw values for representation.

### 2.5 ELISA assays

Plasma levels of ST2, REG3*α*, VCAM1, ICAM1, and TIM3 levels were measured by ELISA according to the manufacturer’s protocol. Plasma samples were diluted (1:50 for ST2, 1:25 for TIM3, 1:10 for REG3*α*, 1:2000 for VCAM1 and 1:500 for ICAM1) as previously reported [18,19] and in consultation with Dr. Sophie Paczesny before the start of ELISA. All biomarkers were measured simultaneously in each patient sample to avoid variation due to freeze-thaw cycles. Absorbance was measured at 450-570 nm using SpectraMax M (Molecular Devices,San Jose, CA, USA). Results were calculated from a 4-parametric logistic curve fit generated using the standards of respective biomarkers. Protein concentrations from individual samples were estimated according to the final dilution factors. ELISAs were performed by investigators who were blinded to all clinical information including transplantation outcomes.

### 2.6 Clinical Endpoints

Neutrophil recovery was defined as an absolute neutrophil count ≥0.5 x 10^9/L for three consecutive days while platelet engraftment was defined as platelet count >20,000/mm^3^ without platelet transfusion for 7 days. Grading and staging of acute and chronic GVHD was done according to standard CIBMTR criteria [20]. Sinusoidal obstruction syndrome (SOS) also called veno-occlusive disease (VOD) was defined as per McDonald’s criteria [21].

### 2.7 Statistical methods

Differences in biomarker levels at different time points between GVHD- and GVHD+ patients and SOS+ and SOS-patients were assessed using Mann-Whitney *U* test. Receiver operating characteristic (ROC) curves and area under the curves (AUCs) were estimated non-parametrically. A *P*-value of <0.05 was considered statistically significant. Differences in cumulative incidences of non-relapse mortality (NRM) and SOS/VOD were calculated by log rank test. Overall survival (OS) was estimated by Kaplan Meier method and the difference between groups were calculated by log rank test. Cumulative incidence analysis was used to describe the association between NRM and biomarkers with relapse as a competing risk. All statistical analysis was carried out using GraphPad Prism version 6.0 (San Diego, CA) and IBM SPSS statistics 24.0 (Armonk, NY). Cumulative analysis was done with R software (version 3.6.1)

## 3 Results

### 3.1 Patient demographics

**Figure 1:**
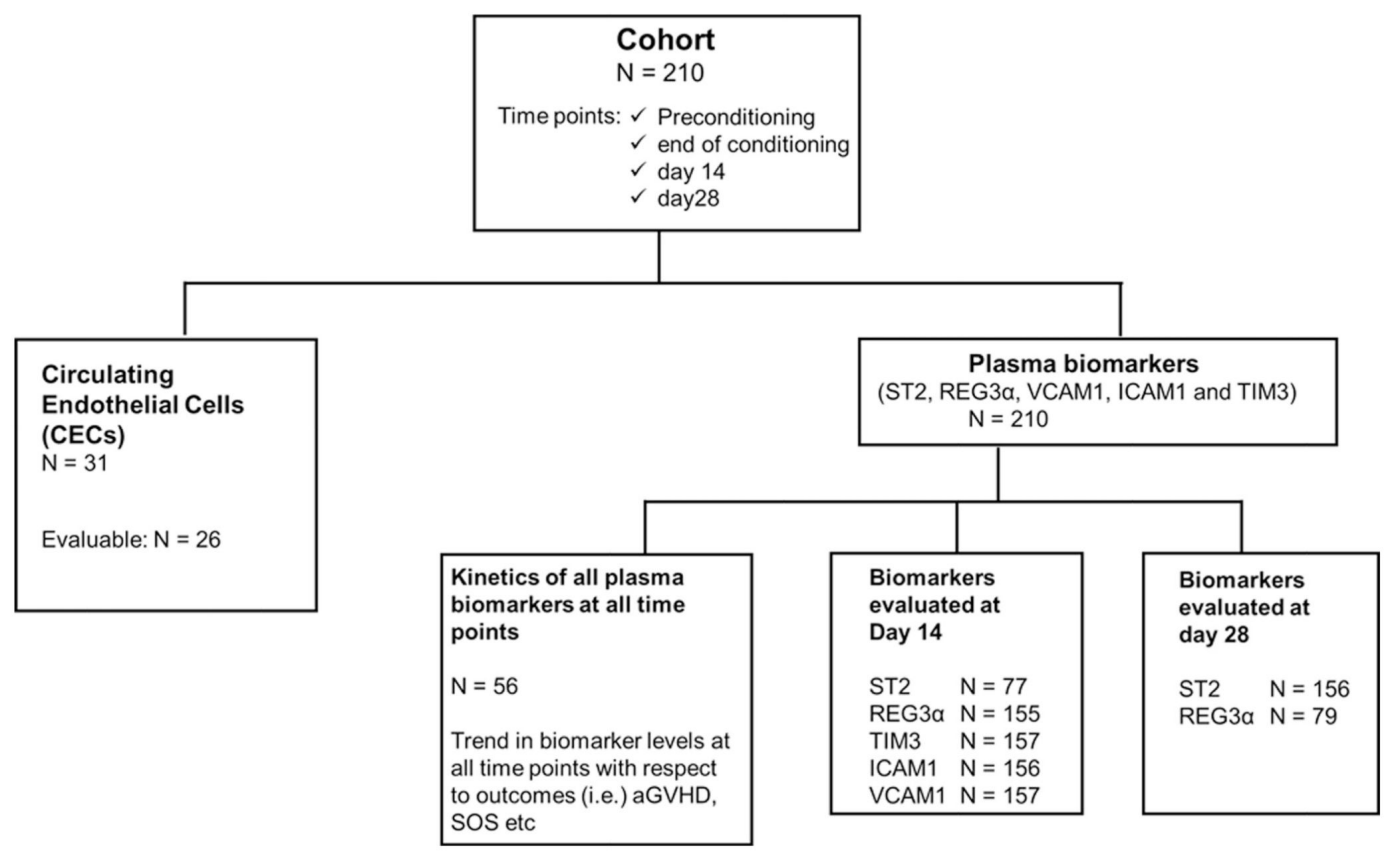
Flowchart illustrating the outline of the study and the number of patients included in each analysis

The median age of patients in which CEC was enumerated was 15 years (range 2-53; 17 males and 9 females; n = 26). Plasma biomarkers were evaluated for all patients enrolled in our study (n = 210). The characteristics of all patients and clinical outcomes post-HSCT are summarized in Table 1. The outline of the study with number of patients in each analysis is summarized in Figure 1.

### 3.2 Kinetics of CEC levels

**Figure 2:**
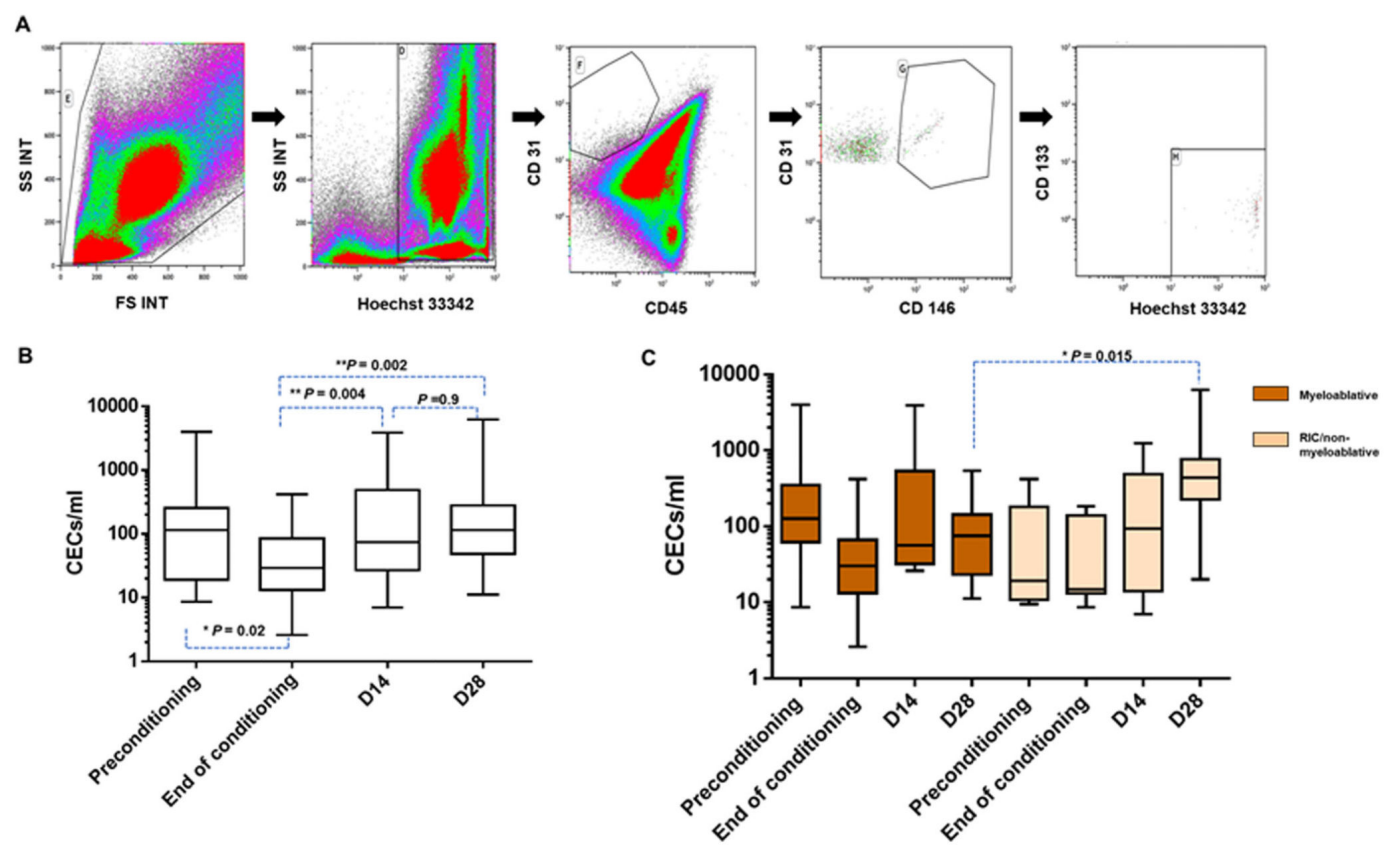
Evaluation of circulating endothelial cells (CECs) as biomarkers for hematopoietic stem cell transplantation (HSCT) complications. A, Gating strategy to evaluate circulating endothelial cells (CECs) based on surface markers Hoechst 33342^+^/CD45^−^/CD31^+^/CD146^+^/CD133^−^. B, Box-whiskers plot showing kinetics of CECs at different time points, that is, preconditioning (n = 27), end of conditioning (n = 26), D14(n = 26), and D28 (n = 26) post HSCT. Boxes show 25th percentile, median and 75th percentile, whiskers show the minimum and maximum values. C, CEC levels between myeloablative conditioning regimen (preconditioning [n = 20], end of conditioning [n = 19], D14 [n = 18], and D28 [n = 19]) and non-myeloablative/reduced intensity conditioning (preconditioning [n = 7], end of conditioning [n = 7], D14 [n = 8], and D28 [n = 7])

Although our original plan was to enumerate CECs at various time points during and after conditioning and post-HSCT, due to technical challenges such as low blood counts and the inability to acquire 1 x 10^6^ events, CECs defined as Hoechst 33342^+^/CD45^−^/CD31^+^/CD146^+^/CD133^−^ (Figure 2A), were evaluated only in 31 out of which enumeration was possible in 26 patients. There was a significant decrease in CEC counts at the end of the conditioning (*P* = .02 vs preconditioning), but showed steady increase at D14 (*P* = .004 vs end of conditioning), and at D28 (*P* = .002 vs end of conditioning) as compared to the baseline counts (Figure 2B and Table 2). An overall comparison of CEC numbers at each time point between myeloablative and reduced intensity conditioning (RIC)/non-myeloablative regimens indicated no significant difference between CECs counts at preconditioning, end of conditioning, and D14. However, at D28 the CEC counts of RIC/non-myeloablative regimens were significantly elevated (*P* = .015) compared to myeloablative conditioning regimen (Figure 2C and Table 2).

When CECs counts at each time points were compared with transplant outcomes, there was elevated CEC counts at the end of the conditioning in patients who developed GVHD compared to those without GVHD, although not statistically significant (*P* = .14). When compared the conditioning regimen intensity between patients who developed GVHD versus those who did not, majority of patients in the GVHD group had received myeloablative regimen (5 out of 8 with GVHD vs 4 out of 15 without GVHD), although not reaching statistical significance. Statistical analysis with SOS/VOD patients could not be performed since there were only two SOS cases in this group.

### 3.3 Longitudinal evaluation of all five plasma biomarkers in a subset of patients (N = 56)

**Figure 3:**
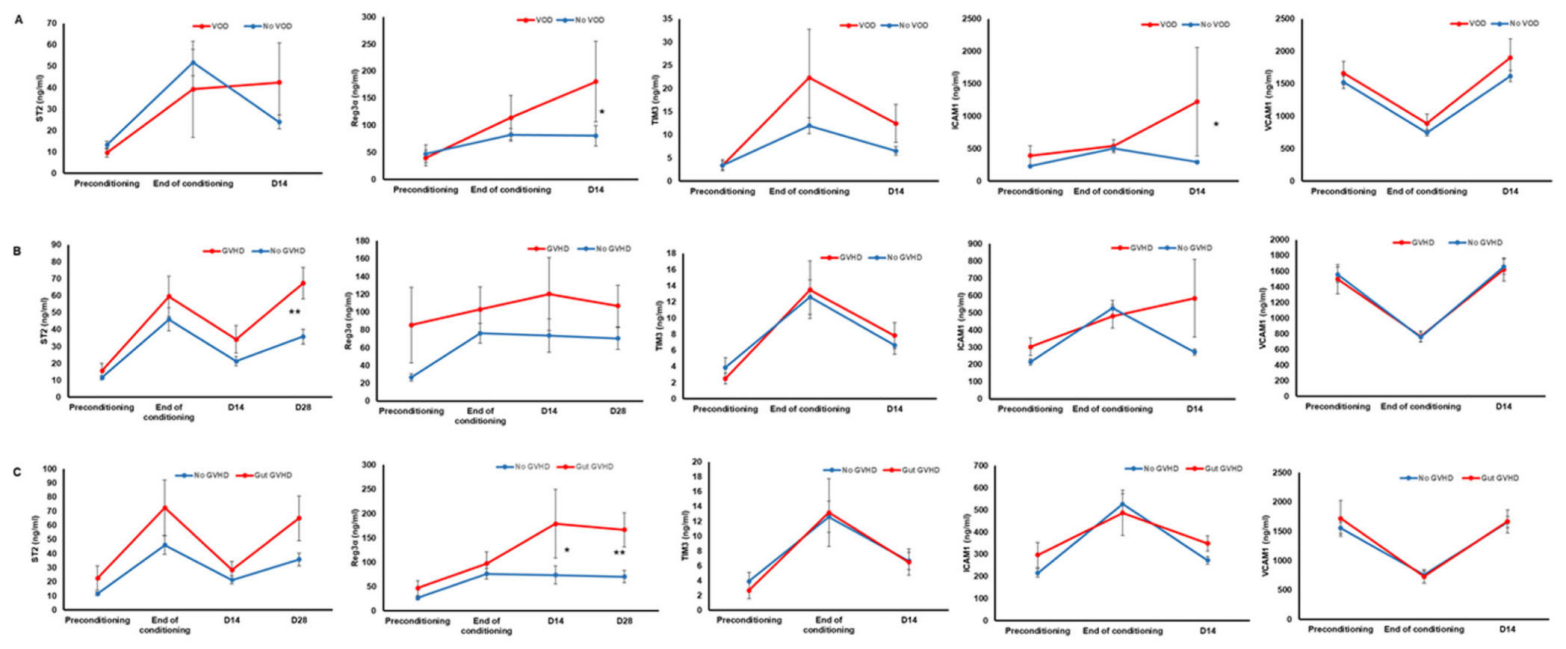
Kinetics of plasma biomarkers (ST2, REG3*α*, TIM3, ICAM1, and VCAM1) in an initial cohort (n = 56) to evaluate the trend in their levels with respect to clinical outcomes. A, kinetic until day 14 for all five biomarkers for patients with VOD (n = 5) and without VOD (n = 51). B, kinetic until day 28 (for ST2 and REG3*α*) or until day14 (for TIM3, ICAM1, and VCAM1) for patients with acute GVHD (n = 19) and without acute GVHD (n = 37). C, kinetic until day 28 (for ST2 and REG3*α*) or until day14 (for TIM3, ICAM1, and VCAM1) for patients with gastrointestinal GVHD (n = 9) and without acute GVHD (n = 37). Mann-Whitney *U* test was used to compare the levels of biomarkers at a specific time point in patients with VOD or without VOD, with aGVHD or without aGVHD and with gastrointestinal GVHD or without aGVHD

Five biomarkers (ST2, REG3*α*, VCAM1, ICAM1, and TIM3) were evaluated longitudinally in an initial subset of 56 patients to determine the trend in their levels across different time points as well as their association with transplant outcomes. The mean levels of four biomarkers (ST2, REG3*α*, VCAM1, ICAM1) appeared to be increased from preconditioning through end of conditioning till day 14 post HSCT. However, the mean levels of VCAM1 were decreased on end of conditioning and increased on day 14. All five biomarkers were consistently elevated at D14 in patients who developed SOS/VOD compared to those without SOS/VOD (Figure 3A). Median day of SOS onset was 5.5 (range: –5 to 11 days). Table S1 shows the median, 25th, and 75th percentiles of biomarker concentrations of all five biomarkers in SOS+ and No SOS groups until D14 post-HSCT with corresponding AUC derived from ROC analysis and *P*-values. Based on these results, we tested all five plasma biomarkers on D14for all patients in our cohort for their potential prognostic values toward SOS/VOD.

Similarly, in patients who developed acute GVHD (all grades included), D28 ST2, and REG3*α* as well as D14 ICAM1 were significantly higher compared to those without GVHD (Figure 3B and Table S2). Median onset day of GVHD was 44 (range: 10-204). A sub analysis of biomarker levels in those who developed gastrointestinal GVHD alone indicated elevated plasma levels of D28 ST2 and D14 REG3*α* and D28 REG3*α* compared to those without GVHD (Figure 3C). Based on these results, we analyzed D28 ST2, D28 REG3*α*, and D14ICAM1 in all patients for their potential prognostic value towards aGVHD.

### 3.4 Plasma biomarkers (REG3*α*,VCAM1, ICAM1, and TIM3) on day 14 associated with SOS/VOD

**Figure 4:**
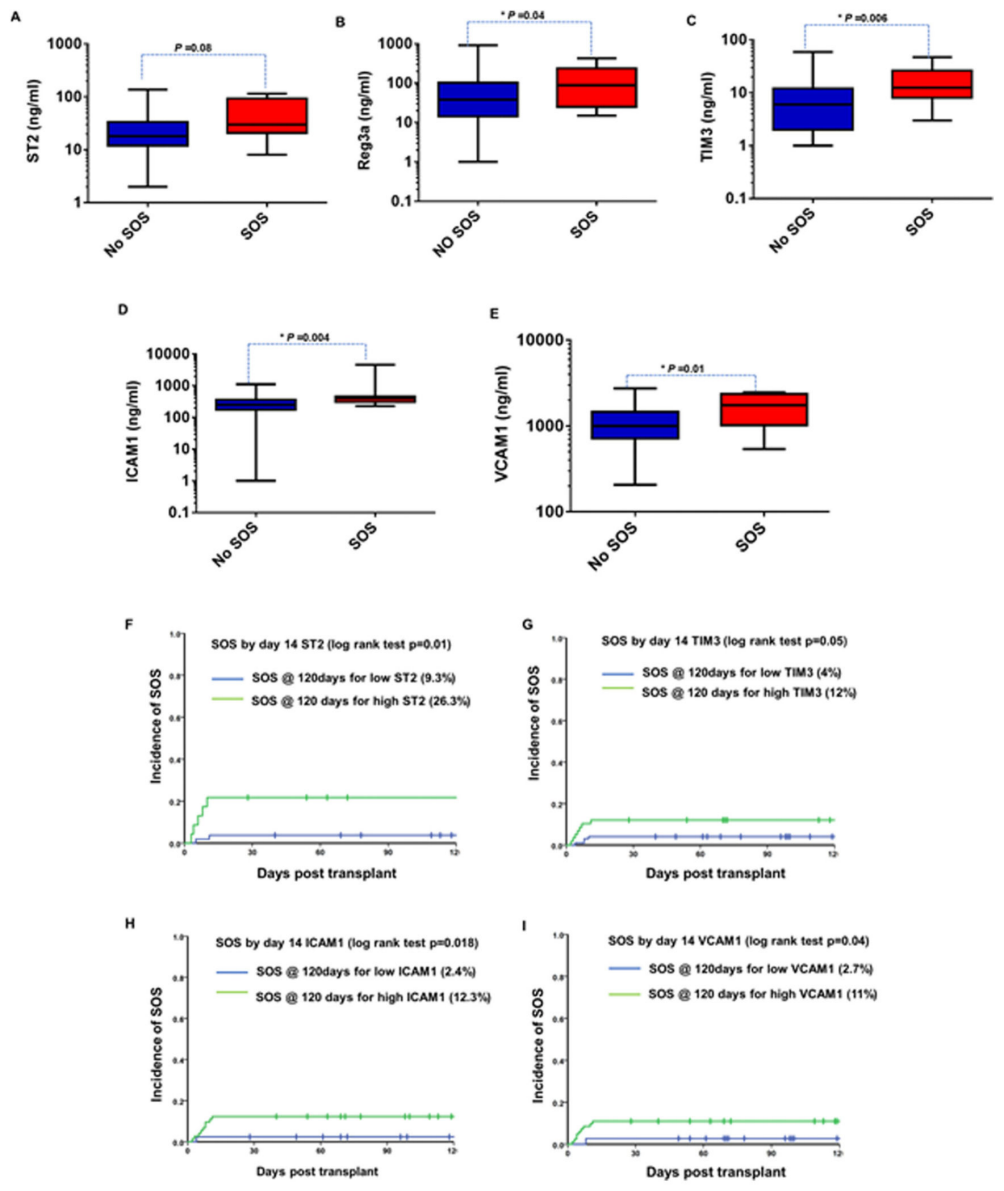
Prognostic value of plasma levels of ST2, Reg3*α*, TIM3, ICAM1, and VCAM1 at D14 toward SOS. A. ST2 concentrations at D14 in patients with SOS (n = 8) and without SOS (n = 69). B, Reg3*α* concentrations at D14 in patients with SOS (n = 11) and without SOS (n = 144). C, TIM3 concentrations at D14 in patients with SOS (n = 12) and without SOS (n = 145). D, ICAM1 concentrations at D14 in patients with SOS (n = 12) and without SOS (n = 144). E, VCAM1 concentrations at D14 in patients with SOS (n = 12) and without SOS (n = 145). Mann-Whitney *U* test was used to compare the levels of biomarkers in patients with SOS and without SOS. Cumulative incidence of SOS based on plasma levels of: F, D14 ST2 with cut off value 29 ng/ml;G, D14TIM3 with cut-off value 8 ng/mL; H, D14 ICAM1 with cut-off value 286 ng/mLand I, D14VCAM1with cut-off value 992 ng/mL

Among 210 patients, plasma samples collected on D14 were available only for 157 patients. The levels of REG3*α*, VCAM1, ICAM1, and TIM3 were significantly elevated at D14in patients who developed SOS compared to those who did not develop SOS (Figure 4A-E). ROC curve analysis for each biomarker at D14 for SOS resulted in an AUC of more than 0.68 (Figure S1). Cut-off values were derived from this analysis, which were further used to stratify samples from patients as those with high or low biomarker values. Table 3 shows the median, 25th, and 75th percentiles of biomarker concentrations of all five biomarkers in SOS+ and SOS-groups at D14 post-HSCT with corresponding AUC, cut-off values, and *P*-values. Based on the cut off values, the cumulative incidence function analyses were performed to predict the likelihood of SOS occurrence based on the biomarker levels. The cumulative incidence of SOS was 26.3% in patients with high ST2, compared to 9.3% in patients with low ST2 (Figure 4F). The cumulative incidence of SOS was 12% in patients with high TIM3, compared to 4% in patients with low TIM3, although not reaching statistical significance (Figure 4G). For patients with high ICAM1, the cumulative incidence of SOS was 12.3%, whereas for patients with low ICAM1, it was 2.4% (Figure 4H). Similarly, for patients with high VCAM1, the cumulative incidence of SOS was 11%, whereas for patients with low VCAM it was 2.7% (Figure 4I)

### 3.5 Plasma biomarker (D28 ST2 and D14ICAM1) levels associated with acute GVHD

**Figure 5:**
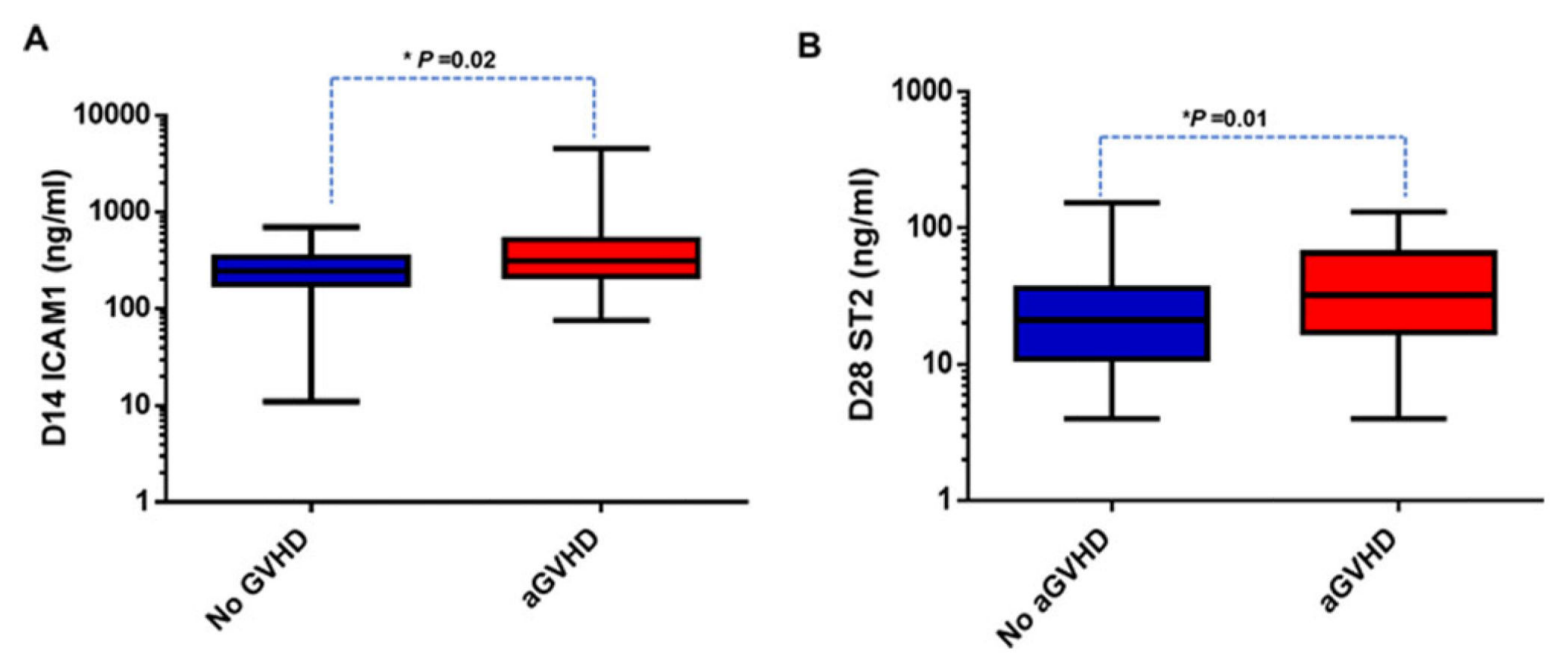
Prognostic value of plasma levels of ST2 at D28 and ICAM1 at D14 toward acute GVHD. A, ICAM1 levels at D14 in patients with aGVHD (n = 47) and without aGVHD (n = 106). B, ST2 levels at D28 in patients with aGVHD (n = 29) and without aGVHD (n = 111). Mann-Whitney *U* test was used to compare the levels of biomarkers in patients with aGVHD and without aGVHD

D14ICAM1 and D28 ST2 levels were significantly elevated in patients who developed acute GVHD (all grades) when compared to those without GVHD (Figure 5). The ROC analysis also demonstrated significant association of D14ICAM1 and D28 ST2 with acute GVHD (AUC of 0.6 and 0.65, respectively; Figure S2). Table 4 shows the median, 25th, and 75th percentiles of biomarker concentrations of D14 ICAM1 and D28 ST2 in acute GVHD+ and acute GVHD-groups with corresponding AUC, cut-off values, and P-values. These analyses were done including samples from patients with the day of GVHD onset as >14 for ICAM1 and >28 for ST2.

**Figure 6:**
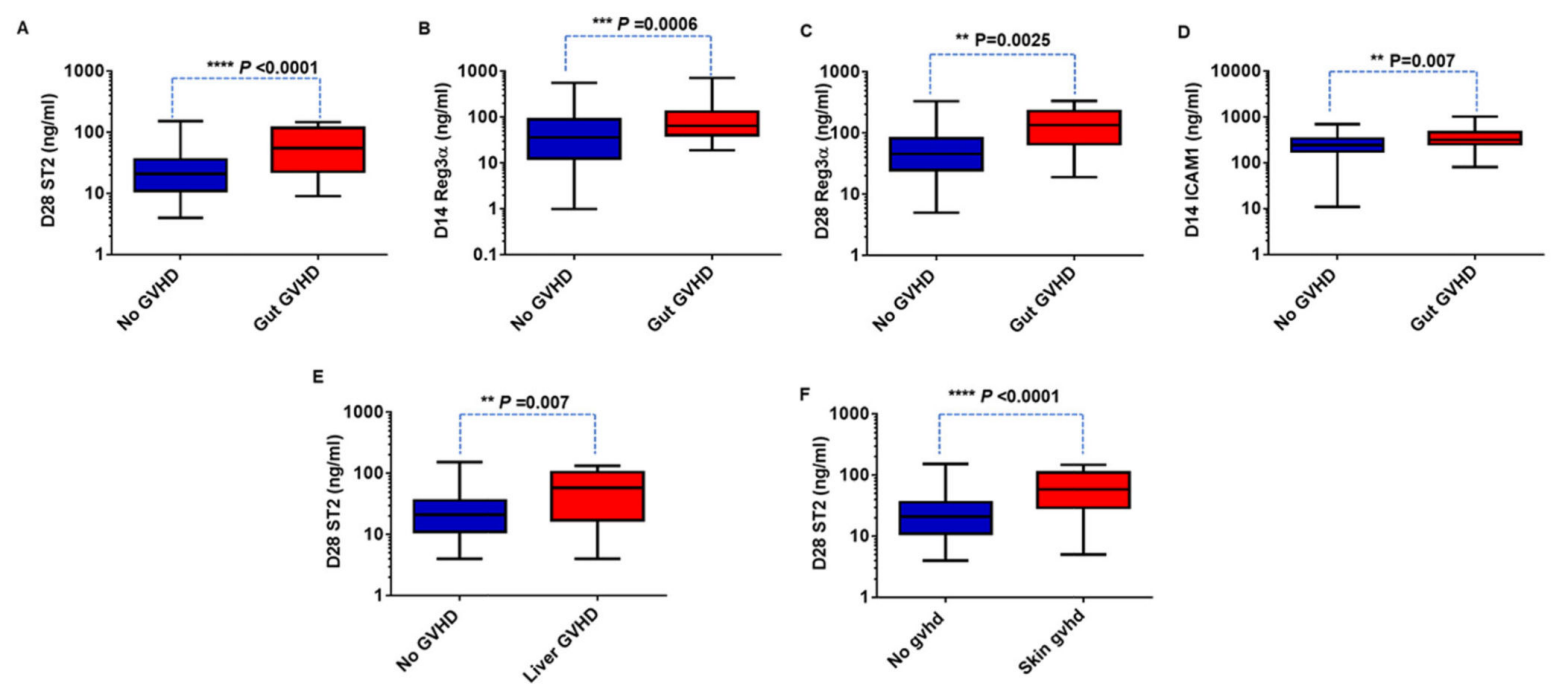
Prognostic value of plasma levels of ST2, Reg3*α*, and at ICAM1 toward organ specific GVHD. A, Reg3*α* concentrations at D14 in patients with gut GVHD (n = 29) and without GVHD (n = 106). B, Receiver operator characteristic (ROC) curve for Reg3*α* at D14 comparing patients with and without gut GVHD. E, ST2 concentrations at D28 in patients with liver GVHD (n = 16) and without GVHD (n = 111). F, ST2 concentrations at D28 in patients with skin GVHD (n = 26) and without GVHD (n = 111). Mann-Whitney *U* test was used to compare the levels of biomarkers in patients with gut/liver/skin GVHD and without GVHD

Upon sub-analysis, D28 ST2, D14 and D28 REG3*α*, and D14ICAM1 levels were significantly elevated in patients who developed gastrointestinal GVHD versus those who did not (Figures 6A-D). The ROC analysis also demonstrated a significant association of these biomarkers at D14 with gastrointestinal GVHD with an AUC of 0.7 (Figure S3). D28 ST2 levels were also significantly elevated in patients who developed liver or skin GVHD versus those who did not develop GVHD (Figure 6E,F). ROC curve analysis of D28 ST2 for liver and skin GVHD resulted in an AUC of more than 0.68 (Figure S4).

### 3.6 Plasma biomarker levels associated with non-relapse mortality and overall survival

**Figure 7:**
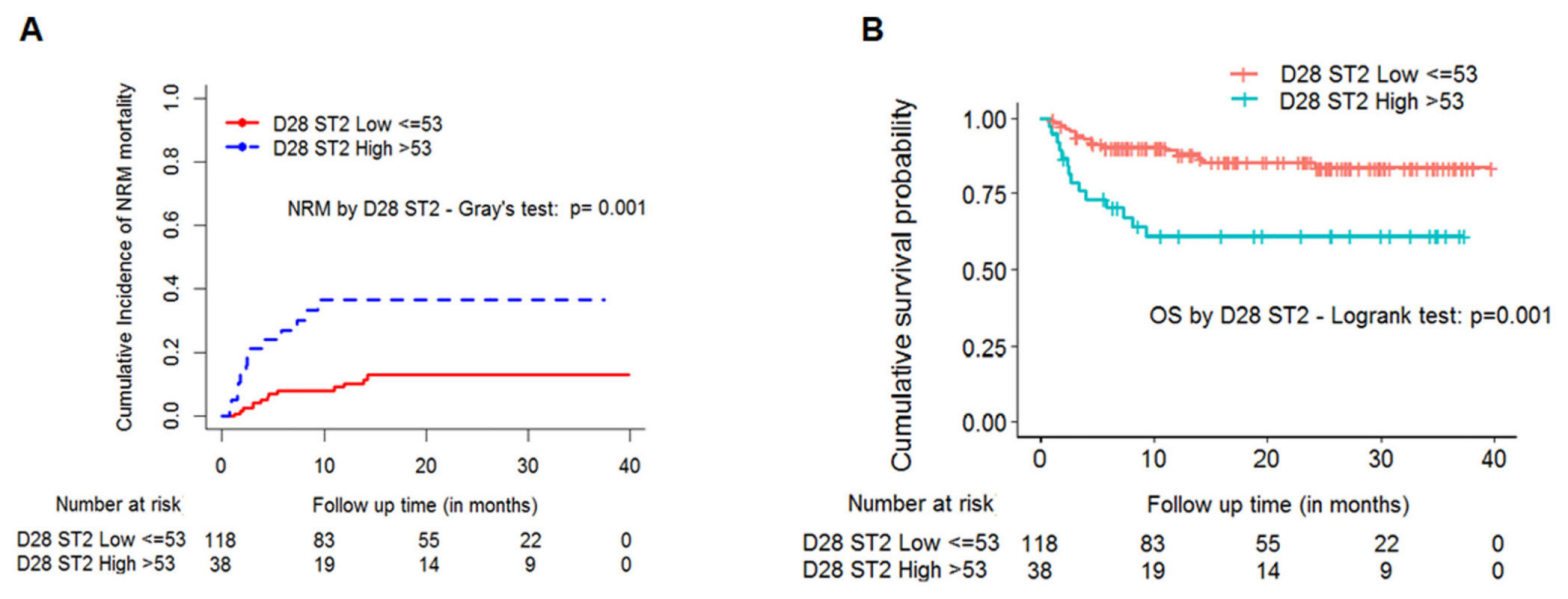
High ST2 levels were correlated with NRM and OS. The cumulative incidence of NRM by 48 months (4 years) stratified by: A, day 28 ST2 levels (high vs low, median cutoff of 53 ng/mL); B, Kaplan-Meier curve for OS stratified by day 28 ST2 levels (high vs low, cutoff of 53 ng/mL)

We then analyzed whether or not these biomarkers predict non-relapse mortality (NRM) or influence overall survival (OS). The incidence of NRM was 34.2% in patients with high ST2 compared with 11% for those with low ST2 values on D28. This was significant when cumulative incidence analysis was done to describe association between D28 ST2 and NRM with relapse as a competing risk (Gray’s test *P* = .001, Figure 7A). The causes of death are included in Table S3. Since D28 ST2 was significantly associated with acute GVHD, we evaluated the role of these biomarkers on 4-year OS based on the cut-off values. The 4-year OS was 63.2% in patients with high ST2 compared to 85.6% for those with low ST2 (*P* = .001, Figure 7B).

## 4 Discussion

Early transplant related complications such as GVHD and RRT influences the success of HSCT, which is the only currently available cure for various hematological disorders. Biomarkers have been evaluated by various groups during and post HSCT as diagnostic/prognostic tool for RRTs and GVHD. [18,8,9,22,2] However, these biomarkers are still being validated across different centers and are not available for routine clinical use. Since an increasing body of evidence suggests a possible role of endothelium in pathophysiology of RRTs and GVHD, in this study, we evaluated cellular (CECs) and soluble plasma biomarkers (VCAM1, ICAM1) to assess the level of endothelial damage and to evaluate their potential diagnostic/prognostic value as biomarkers for GVHD and early transplant related complications in patients undergoing HSCT. We have included previously validated biomarkers such as ST2, REG3*α*, and TIM3 to further validate them in patients who underwent HSCT in our center.

Upon longitudinal evaluation of CECs until D28 post HSCT, we observed an approximately four fold increase in median CEC counts in patients who developed GVHD compared to those without GVHD at the end of the conditioning. This is in contrast with previous reports, [8,11] where increased CEC levels were reported in patients without GVHD compared to those with GVHD at the end of conditioning and at engraftment. This could be explained by the different methodology used (CellSearch System) and the immunophenotypic definition of CECs defined as CD146^+^/CD105^+^/DAPI^+^/CD45^−^. Furthermore, CECs could be influenced by multiple factors such as conditioning regimen [9,23], GVHD prophylaxis [8, 9], immunosuppressive treatments [24, 25], and infections [8]. However, our observation of elevated CEC levels at the end of conditioning in patients who developed GVHD was not statistically significant, probably due to the small numbers evaluated. A potential limitation of enumerating CECs is the relatively low numbers of blood cells in circulation post-conditioning that makes acquisition of rare CECs cumbersome. Also, lack of consensus on the immunophenotypic definition of CECs and lack of standardized methodology to enumerate them, makes validation of CECs as biomarkers across different centers challenging.

We observed all five plasma biomarkers that we measured to be significantly elevated on D14 in patients with SOS. While the potential prognostic significance of ST2 [18], VCAM1 [18], and ICAM1 [26] toward SOS is consistent with previous reports, association of two other biomarkers (ie, endothelial expressed TIM3 and Paneth cell expressed REG3*α*) with SOS is a novel observation. Although it is well known that endothelial injury/dysfunction triggers pathogenesis of SOS, it is not clear whether this observation is due to the increased levels of individual biomarkers or the cumulative effect of all the biomarkers that are responsible for this prognostic significance.

Elevated D28 ST2 and D14 ICAM1 levels associated significantly with acute GVHD in this study thus explaining the possible prognostic value of these biomarkers in HSCT. These analyses were done by considering samples with days of GVHD onset as > 14 for ICAM1 and > 28 for ST2, which improves the possible predictive values of these biomarkers toward likelihood of GVHD occurrence. Moreover, we have included patients with all grades of GVHD and organs of GVHD involvement. Upon sub-analyses, we observed a significant association of D28 ST2 with gastrointestinal, liver, and skin GVHD and D14/D28 REG3*α*, D14ICAM1 with gastrointestinal GVHD. This is interesting since most of the previous reports have measured biomarkers either at the onset of GVHD or at initiation of GVHD treatment. In addition, we observed that high ST2 at D28 was strongly associated with NRM. High plasma biomarker levels (such as D28 ST2) associated with GVHD and NRM could pave way for risk stratifying patients for therapy. This is consistent with previous reports where high ST2 levels on D28 either alone [18] or as a panel of biomarkers (ST2, REG3*α*, and TNFR1) [27,28] were demonstrated to be predictive of GVHD and NRM. Thus, the predictive values of these biomarkers could potentially aid clinicians in assessing and managing HSCT complications effectively.

To the best of our knowledge, this is the first report where we have evaluated biomarkers comprehensively in HSCT patients and identified biomarkers that can predict early complications arising due to HSCT. While we derived cut-off values for these biomarkers to risk stratify patients for HSCT complications, this need to be validated in an independent cohort or in large multicenter trials for future clinical applications. Moreover, based on the cut-off values, it is possible to define a high-risk population that would benefit from a pre-emptive intervention that will need still to be defined (eg, cortecosteroids at 1 mg/kg/day for 7-10 days with rapid taper). The major limitation of this study is the significant overlap in biomarker levels between groups with and without HSCT related complications. Further, many groups have reported several cut-off values for ST2 (33.9 ng/mL [18]; 740pg/mL [29]; 3230 ng/mL [30]) and REG3*α* (151 ng/mL [19]; 1989 pg/mL [30]) which makes establishing reproducible cut-off values for biomarkers a challenge. We also believe evaluating the biomarkers prospectively at this and earlier time points will better validate the association of these biomarkers with outcomes and could potentially explain their predictive values.

## Supplementary Material

Additional supporting information may be found online in the Supporting Information section at the end of the article

Supplementary figure

## Figures and Tables

**Table 1:** Characteristics of patients evaluated for cellular and plasma biomarkers

Characteristics		N = 210 (%)
Age	median (range)	13.5(1-65)

Sex	Male	138 (66)
	Female	72 (34)

Disease	AML	43 (20)
	MDS	19(9)
	ALL	20 (10)
	Acute biphenotypic leukemia	1 (0.5)
	CML	5(2)
	CLL	1 (0.5)
	AA	25 (12)
	FA	11 (5)
	Thal	83 (40)
	Adrenoleukodystrophy	1 (0.5)
	Pure Red cell aplasia	1 (0.5)

Donor	Related	178 (85)
	unrelated	32 (15)

Conditioning regimen intensity	Myeloablative	169 (80)
	Non-myeloablative	35 (17)
	Reduced intensity	6(3)

SOS/VOD	yes	18 (9)
	No	192(91)

Acute GVHD	Yes	69 (33)
	No	141 (67)

Chronic GVHD	Yes	51 (24)
	No	159(76)

Mucositis	Grade 3	83 (40)
	Grade 2	77 (37)
	Grade 1	16(8)
	No mucositis	34 (16)

NRM	Yes	19(9)
	No	191 (91)

**Table 2:** Median and range of CEC counts across all time points

Biomarker	Preconditioning Median (range)	End of conditioning Median (range)	D14 Median (range)	D28 Median (range)
Circulating Endothelial cells (CECs)-all conditioning regimen	114(9-4000)	25(3-420)	74(7-3914)	115(11-6300)
Myeloablative conditioning	126(9-4000)	30(3-420)	57(26-3914)	75(11-540)
Non-myeloablative/reduced intensity conditioning	11(9-420)	15(9-184)	93(7-1240)	438(20-6300)

**Table 3:** Median concentrations and AUCvsVODfor 5 biomarkers at D14

Biomarker	NoVOD (ng/mL) Median (25th percentile, 75th percentile)	VOD (ng/mL) Median (25th percentile, 75th percentile)	Cut-off value	ROC AUC (95% CI)	*P*-value
ST2	18(12,34)	30 (21, 94)	29 ng/mL	0.68 (0.59-0.78)	.04
ICAM1	251(174,360)	398 (309,454)	286 ng/mL	0.75 (0.65-0.83)	<.001
REG3*α*	38 (14,103)	88 (25, 237)	66 ng/mL	0.69 (0.54-0.83)	.04
VCAM1	999 (715,1462)	1757(1023,2348)	992 ng/mL	0.72 (0.62-0.8)	.002
TIM3	6 (2,12)	13(8, 26)	8 ng/ml	0.73 (0.64-0.82)	.001

**Table 4:** Median concentrations and AUC versus aGVHDfor biomarkers

Biomarker	Time point	NoGVHD Median (25th percentile, 75th percentile)	Acute GVHD Median (25th percentile, 75th percentile)	Cut-off value	ROC AUC (95% CI)	*P*-value
ST2	D28	21(11,36)	32(17,65)	53 ng/ml	0.65 (0.53-0.76)	.01
ICAM1	D14	245(177,340)	312(215,517)	356 ng/ml	0.6(0.51-0.7)	.02

## Key points

High ST2 levels on Day28 post HSCT predicts acute GVHD, non-relapse mortality (NRM) and overall survival in HSCT patients

First study from India to comprehensively evaluate plasma biomarkers predictive of early complications post HSCT
