# Supplementary figures and images for: Prognostic plasma biomarkers of early complications and graft‐versus‐host disease in patients undergoing allogeneic hematopoietic stem cell transplantation
Source: EJHaem. 2020 Jun 17;1(1):219–29. doi: 10.1002/jha2.26 (PMC7116009; doi:10.1002/jha2.26)

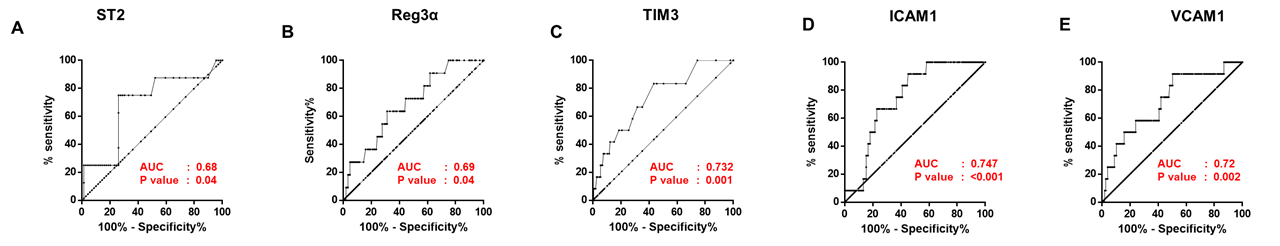

Supplement: Supplementary file 1 — Supporting information [file JHA2-1-219-s003.tif]

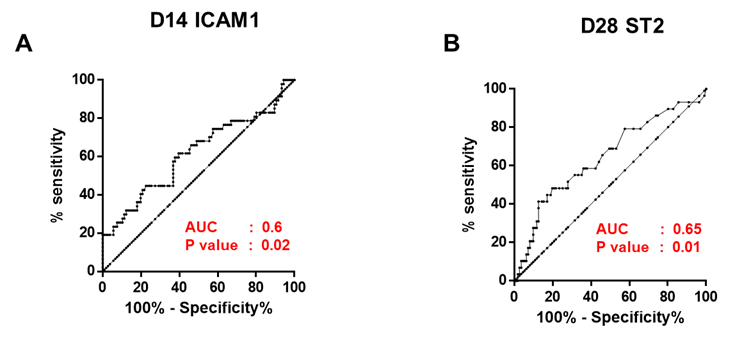

Supplement: Supplementary file 2 — Supporting information [file JHA2-1-219-s005.tif]

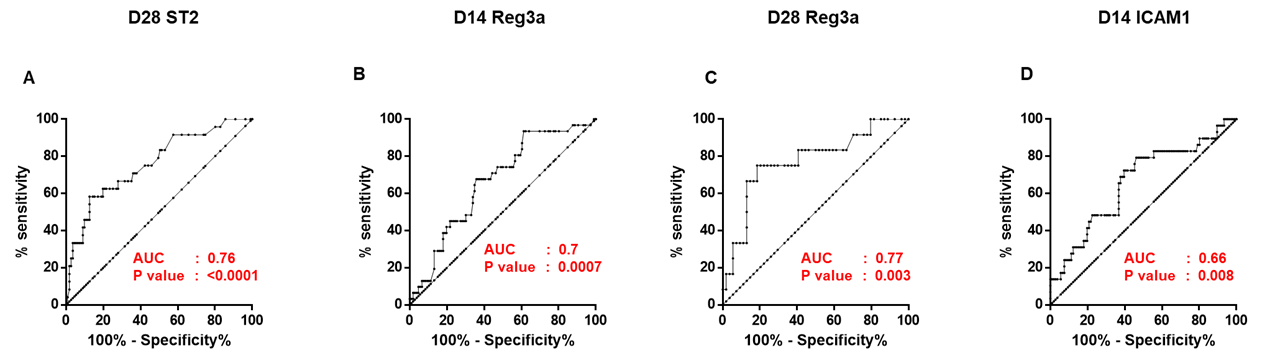

Supplement: Supplementary file 3 — Supporting information [file JHA2-1-219-s002.tif]

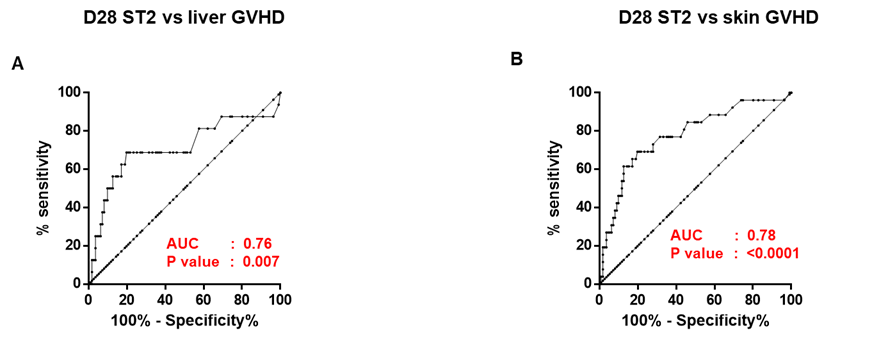

Supplement: Supplementary file 4 — Supporting information [file JHA2-1-219-s001.tif]
