# Supplementary material for: Prognostic plasma biomarkers of early complications and graft‐versus‐host disease in patients undergoing allogeneic hematopoietic stem cell transplantation
Source: EJHaem. 2020 Jun 17;1(1):219–29. doi: 10.1002/jha2.26 (PMC7116009; doi:10.1002/jha2.26)
Supplement: Supplementary file 5 — Supporting information [file JHA2-1-219-s004.docx]

**Supplementary information**

**Table S1**


**Table S2**

**Table S3: Cause of death**

| **Mortality causes** | **Total cohort where D28 ST2 evaluated**  **N=156** |
| --- | --- |
| Relapse/progressive disease | 8 (5%) |
| GVHD | 12 (8%) |
| Sepsis/Infection | 6 (4%) |
| Poor graft function | 3 (2%) |
| Graft failure | 1 (0.6%) |

**Supplementary Figure legends:**

Fig S1. Receiver operator characteristic (ROC) curve and area under the curve (AUC) for ST2, REG3α, TIM3, ICAM1 and VCAM1 at D14 respectively, comparing patients with and without SOS.

Fig S2. Receiver operator characteristic (ROC) curve and area under the curve (AUC) for ICAM1 at D14 and ST2 at D28 comparing patients with and without aGVHD.

Fig S3. Receiver operator characteristic (ROC) curve and area under the curve (AUC) for D28 ST2, D14/D28 Reg3α and D14 ICAM1 comparing patients with gastrointestinal GVHD and without aGVHD.

Fig S4. Receiver operator characteristic (ROC) curve and area under the curve (AUC) for D28 ST2 comparing patients with liver or skin GVHD and without aGVHD.
