# Supplementary material for: Prognostic plasma biomarkers of early complications and graft-versus-host disease in patients undergoing allogeneic hematopoietic stem cell transplantation
Source: EJHaem. Author manuscript; Available in PMC 2020 Oct 1. (PMC7116009; doi:10.1002/jha2.26)
Supplement: Supplementary file 1 [file EMS88137-supplement-Supplementary_figure.pdf]

# Fig S1

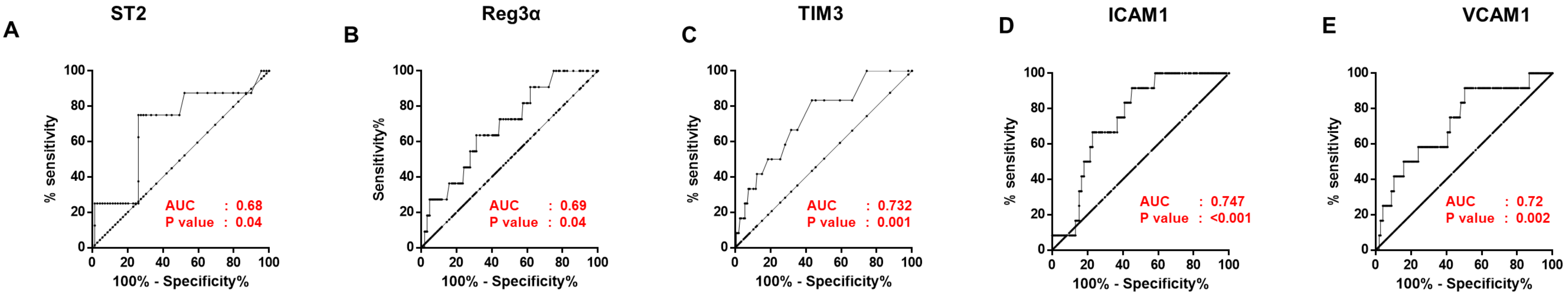
A
ST2
% sensitivity
100% - Specificity%
AUC : 0.68
P value : 0.04
B
Reg3α
Sensitivity%
100% - Specificity%
AUC : 0.69
P value : 0.04
C
TIM3
% sensitivity
100% - Specificity%
AUC : 0.732
P value : 0.001
D
ICAM1
% sensitivity
100% - Specificity%
AUC : 0.747
P value : <0.001
E
VCAM1
% sensitivity
100% - Specificity%
AUC : 0.72
P value : 0.002

## Fig S2

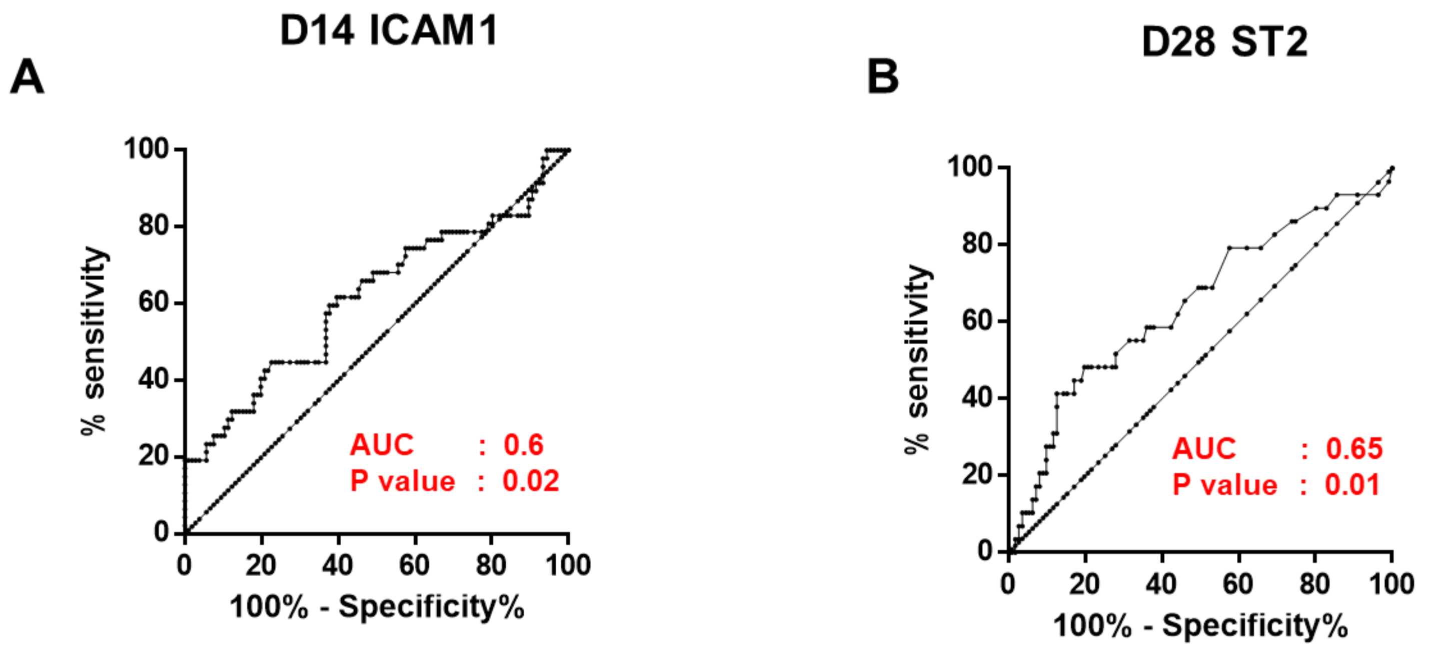
A
D14 ICAM1
% sensitivity
100% - Specificity%
0
20
40
60
80
100
AUC : 0.6
P value : 0.02
B
D28 ST2
% sensitivity
100% - Specificity%
0
20
40
60
80
100
AUC : 0.65
P value : 0.01

## Fig S3

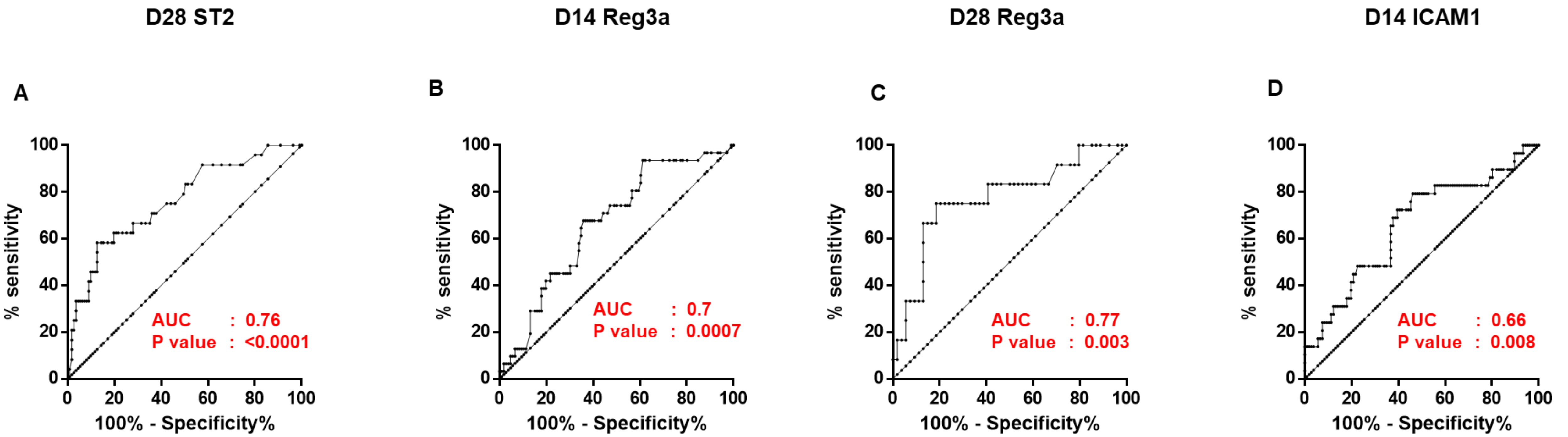
D28 ST2
D14 Reg3a
D28 Reg3a
D14 ICAM1
A
B
C
D
% sensitivity
100% - Specificity%
0
20
40
60
80
100
AUC : 0.76
P value : <0.0001
AUC : 0.7
P value : 0.0007
AUC : 0.77
P value : 0.003
AUC : 0.66
P value : 0.008

**Fig S4**

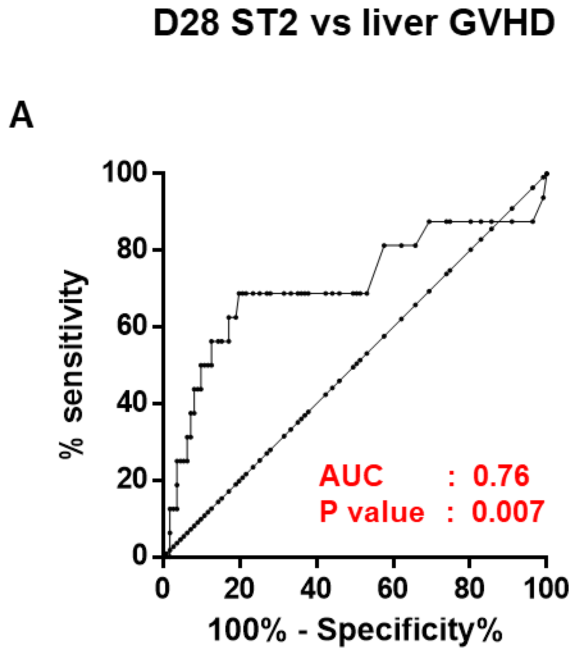
D28 ST2 vs liver GVHD
A
100
80
60
40
20
0
% sensitivity
0
20
40
60
80
100
100% - Specificity%
AUC : 0.76
P value : 0.007

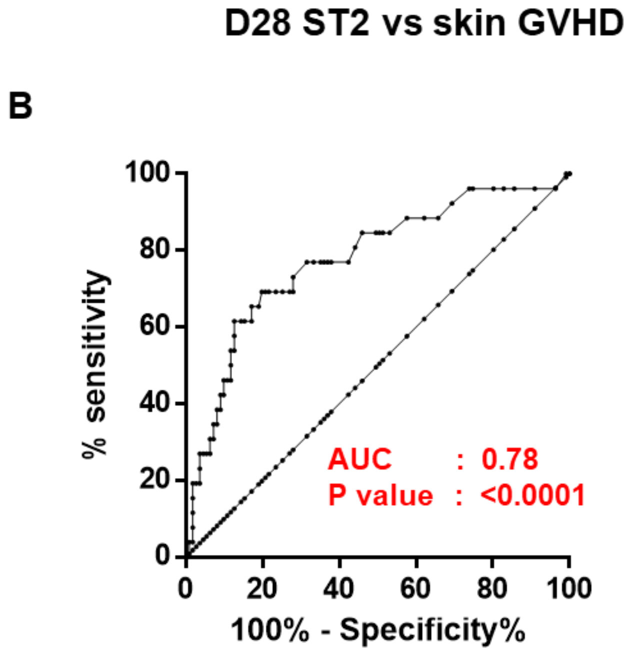
D28 ST2 vs skin GVHD
B
100
80
60
40
20
0
% sensitivity
0
20
40
60
80
100
100% - Specificity%
AUC : 0.78
P value : <0.0001
